# Non‐amnesic preclinical traits in autosomal dominant Alzheimer's disease: Executive functions in *APP*_V717I_ Mexican carriers

**DOI:** 10.1002/alz70857_105651

**Published:** 2025-12-25

**Authors:** Angélica Zuno‐Reyes, Karina Pérez‐Rubio, Ricardo Jáuregui Torres, Sofia Dumois‐Petersen, Luis E Figuera, John M Ringman, Esmeralda Matute

**Affiliations:** ^1^ Instituto de Neurociencias, CUCBA, Universidad de Guadalajara, Guadalajara, JA, Mexico; ^2^ Universidad de Guadalajara, Guadalajara, JA, Mexico; ^3^ Instituto Mexicano del Seguro Social, Guadalajara, JA, Mexico; ^4^ Alzheimer's Disease Research Center, Keck School of Medicine, University of Southern California, Los Angeles, CA, USA; ^5^ Departamento de Estudios en Educación, CUCSH, Universidad de Guadalajara, Guadalajara, JA, Mexico

## Abstract

**Background:**

**We aimed to assess memory and executive functions (EF) performance in preclinical carriers (PC) of** *APP* **_V717I_ autosomal dominant Alzheimer's disease (ADAD) and to know if age and years of schooling (YoS) affect their results**.

**Method:**

**Our research included fifteen PC (** *M* **age = 37.27 years, standard deviation [SD] = 6.82;** *M* **YoS = 10.07 years, SD = 4.10) and twenty‐four non‐carriers (NC;** *M* **age = 32.17 years, SD = 10.95;** *M* **YoS = 9.08, SD = 2.92) of** *APP* **_V717I_, all of them directly family related. We assessed PC and NC groups in a small town in Jalisco, Mexico. All the participants obtained a Clinical Dementia Rating (CDR) score of zero. The EF and memory tasks are included in the Mexican standardization of the neuropsychological protocol from the Consortium to Establish a Registry for Alzheimer's Disease and its complementary tasks (CERAD‐MX; Zuno‐Reyes et al., 2023). We compared PC and NC scores with t‐tests. Finally, we analyzed age and YoS effects through correlations and linear regressions in those comparisons with medium or large effect sizes**.

**Result:**

PC obtained fewer Conceptual Level Responses (CLR; *M* = 16.73, SD = 6.11) than NC (*M* = 22.04, SD = 10.04) on the MWCST. As for the memory tasks, we found similar scores in both PC and NC. Age did not predict this outcome (b = ‐.33, *r* ^2^ = .14, *p* = .175) but showed a moderate negative correlation (*r* = ‐.37, *p* = .175). YoS yielded a strong positive correlation (*r* = .60, *p* = .018) and predicted this result (b = .90, *r* ^2^ = .31, *p* = .018).

**Conclusion:**

**While memory problems are frequently reported in preclinical carriers of other ADAD mutations, our PC‐** *APP* **_V717I_ and NC groups scored similarly on memory tasks. However, we found preclinical difficulties in the MWCST, which assesses cognitive flexibility. YoS is positively correlated and predicts this result. To our knowledge, no published studies report preclinical EF performance in any of the** *APP* **mutations, so our work marks a precedent in exploring EF in** *APP* **V717I through this stage, being this the most frequently identified** *APP* **mutation worldwide**.

